# Evaluating a Method to Estimate Mediation Effects With Discrete-Time Survival Outcomes

**DOI:** 10.3389/fpsyg.2019.00740

**Published:** 2019-04-05

**Authors:** Amanda Jane Fairchild, Chao Cai, Heather McDaniel, Dexin Shi, Amanda Gottschall, Katherine E. Masyn

**Affiliations:** ^1^Department of Psychology, University of South Carolina, Columbia, SC, United States; ^2^Curry School of Education, University of Virginia, Charlottesville, VA, United States; ^3^Department of Population Health Sciences, Georgia State University, Atlanta, GA, United States

**Keywords:** event history, discrete-time, survival analysis, mediation, onset

## Abstract

The utility of evaluating mediation effects spans across research domains. The model facilitates investigation of underlying mechanisms of event timing and, as such, has the potential to help strengthen etiological research and inform intervention work that incorporates the evaluation of mediating variables. In order for the analyses to be maximally useful however, it is critical to employ methodology appropriate for the data under investigation. The purpose of this paper is to evaluate a regression-based approach to estimating mediation effects with discrete-time survival outcomes. We empirically evaluate the performance of the discrete-time survival mediation model in a statistical simulation study, and demonstrate that results are functionally equivalent to estimates garnered from a potential-outcomes framework. Simulation results indicate that parameter estimates of mediation in the model were statistically accurate and precise across the range of examined conditions. Type 1 error rates were also tolerable in the conditions studied. Adequate power to detect effects in the model, with binary X and continuous M variables, required effect sizes of the mediation paths to be medium or large. Possible extensions of the model are also considered.

## Introduction

Mediation hypotheses have become increasingly pervasive across a wide range of research areas in recent years, as investigators use statistical mediation analysis to elucidate developmental pathways of behavior (e.g., Selig and Preacher, 2009; George et al., 2014; Gonzalez and MacKinnon, 2018), inform etiological underpinnings of disease symptoms and other health behaviors (e.g., Ranby et al., 2009, 2011), and enhance the evaluation of prevention/intervention programs (e.g., Petrosino, 2000; Donaldson, 2001; Fairchild and MacKinnon, 2014; Fairchild and McDaniel, 2017). In reaction to this sustained interest in use, methodologists have developed and analyzed a wide array of techniques to examine statistical mediation across a variety of data types, as well as have increased investigations into improving causal interpretation of model parameters. The use of discrete-time survival analysis to model time-to-event occurrence has also increased with applications across a wide range of developmental and behavioral outcomes such as youth alcohol and other drug involvement onset, first relapse following drug or alcohol treatment, early initiation of sexual activity, school expulsion, and timing of death (e.g., Capaldi et al., 1996; Nichols et al., 1999; Xie et al., 2005; Petras et al., 2011;Kerr et al., 2015; Atherton et al., 2016; Beydoun et al., 2016; Mason et al., 2017; Kilburn et al., 2018). To date, however, little methodological work has been conducted to study methods appropriate for examining mediation effects with discrete-time survival outcomes. This is a salient outstanding need.

Previous work has considered the examination of mediation effects with *continuous* time survival data (e.g., Tein and MacKinnon, 2003; Lange and Hansen, 2011; Tchetgen Tchetgen, 2011; Gelfand et al., 2016). The estimation and interpretation of model parameters across continuous vs. discrete-time survival analyses differ in critical ways, however, engendering the need to understand and develop discrete-time survival methods in their own right. These differences manifest in large part due to the “tied survival data” (e.g., Chalita et al., 2002) that materializes when one measures time-to-event occurrence in discrete epochs such as days, months, semesters, or years. Such periodic data collection, which is routine in academic settings and clinical trials, allows multiple individuals to experience an event within the same period, thus yielding grouped survival times. The ensuing focus of analysis in discrete-time models centers on the conditional probability of an individual experiencing the event of interest in a given time period, given that they have not experienced the event prior to that time (i.e., the hazard probability; Masyn, 2014). This focus is in contrast to an emphasis on the usual hazard rate in continuous-time survival models (e.g., Cox, 1972).

Fairchild et al. (2015) discussed key strengths of investigating mediation effects in discrete-time survival data with respect to ameliorating the nationwide public health problem of substance use in youth. Indeed, a brief survey of recent applied work suggests that there is a clear and present interest in examining mediation effects with discrete-time survival outcomes^1^, particularly with research questions relating to different facets of substance use onset in youth. For example, Atherton et al. (2016) examined mediation effects in discrete-time survival data in a sample of Mexican-Origin youth to understand how familism impacted timing to onset of substance use through three proposed mediators: access to substances, intent to use substances, and association with deviant peers. In another study related to timing to substance use initiation, Mason et al. (2017) explored how cumulative contextual risk at the time of birth predicted timing to onset of substance use through childhood peer marginalization, aggression, and behavior problems. Kerr et al. (2015) also examined a substance use model that considered how parental use of marijuana in adolescence impacted time to onset of child marijuana use indirectly via a variety of risk and protective factors at both the child and parent-level. There have been applications beyond the substance use literature as well (Reis et al., 2011; Garner and Hunter, 2013; Hill et al., 2013; Lambert et al., 2013; Slater and Henry, 2013; Beydoun et al., 2016).

Varied tests of mediation were used throughout these applications. Although a handful of examples used contemporary, recommended approaches to test for mediation (e.g., bias-corrected bootstrapped confidence intervals, and potential outcomes approach), the majority of examples employed methods that have been shown to be limited in a variety of circumstances (see MacKinnon et al., 2002; Fritz and MacKinnon, 2007). That is, some of the applications used a point estimate and normal theory standard error estimator to test mediation but did not have sufficient sample size to invoke asymptotic efficiency (Sobel, 1982). Other studies assessed mediation via the causal steps (Baron and Kenny, 1986) and did not report a point estimate of the mediated effect, precluding tests of its statistical significance. An abundant amount of methodological work has demonstrated serious shortcomings with the latter approach to testing mediation effects (e.g., Shrout and Bolger, 2002; MacKinnon and Fairchild, 2009). Namely, the causal steps approach as originally described lacks sufficient power to detect mediation effects in a variety of circumstances (e.g., MacKinnon et al., 2002). This problem is particularly serious in *complete* mediation models, where (in the population) there is no direct effect of an independent variable on the outcome while controlling for the mediator. Indeed, Fritz and MacKinnon (2007) illustrated that the causal steps required approximately *n* = 21,000 subjects for adequate power to detect mediation when component paths of the mediated effect were small in size and the direct effect was zero. Others have also written on issues of power associated with this requirement of the test (e.g., Kenny and Judd, 2014; O’Rourke and MacKinnon, 2015)^2^.

Taken as a set, the above examples serve to highlight the need for well-tested, uniform methods for estimating, and testing mediation effects on discrete time survival outcomes in the applied literature. Fairchild et al. (2015) introduced a framework to test mediation hypotheses with univariate discrete time survival data (i.e., non-recurring events) that incorporates current methodological recommendations from the broader mediation analysis literature. The authors demonstrated both a regression-based approach to analysis, as well as a structural equation modeling-based approach that estimates model parameters within a mixture-modeling framework. Neither variant, however, has yet to be evaluated statistically. Given the utility of examining mediation hypotheses with discrete-time survival outcomes, and the current lack of guidelines to do so, the formal evaluation of such a method is merited.

## Current Study

Our goal in this paper is to evaluate statistical properties of the discrete-time survival mediation (*DTSM*) model. Specifically, we examine the models in a statistical simulation study to examine power, Type 1 error, and accuracy of model parameter estimates. Additionally, in line with an increased focus on causal frameworks to conduct statistical mediation analysis, we demonstrate analytical equivalency (under linearity assumptions) of the Fairchild et al. (2015) approach to that of Imai et al. (2010) potential outcomes framework for estimating the average causal mediated effect (*ACME*) and illustrate that simulation results of the two approaches are functionally indistinguishable. The intention of this study is to document the statistical properties of the DTSM model and to develop recommendations for its use in applied research. To best achieve study goals, we first orient readers to the DTSM model (for a more comprehensive overview of the model and component parts see Fairchild et al. (2015). We then briefly explain the potential outcomes framework for examining mediation effects, before describing the design, and findings of the statistical simulation study. We end by providing general recommendations and considerations for applied researchers, as well as by discussing limitations of our study.

## Discrete-Time Survival Mediation Analysis

Mediation analysis allows a researcher to examine how one or more intermediate variables conveys the effect of a predictor to an outcome of interest, enhancing one’s understanding of theoretical relations in a model (Judd and Kenny, 1981; Baron and Kenny, 1986; MacKinnon et al., 2007a; MacKinnon, 2008). Discrete-time survival analysis supports research questions regarding both the “if” and “when” of an outcome by modeling the probability of event occurrence over discrete intervals of time (e.g., Allison, 1982; Singer and Willett, 1991; Singer and Willett, 1993; Willett and Singer, 2004; Masyn, 2014). This method lends insight into temporal windows of critical risk by providing information on risk for event occurrence in each interval. Drawing on mediation analysis and discrete-time survival analysis, the DTSM model allows testing of hypotheses about the mechanisms of event timing.

In line with conventional mediation models, the DTSM model parses the overall effect of an independent variable on the outcome (here the hazard probability of event occurrence) into a direct effect and an indirect effect. The direct effect in the DTSM model captures the influence of a predictor on the hazard probability of event occurrence controlling for the mediator, and the indirect effect captures the influence of the predictor on the hazard probability of event occurrence through the mediator.

**FIGURE 1:**
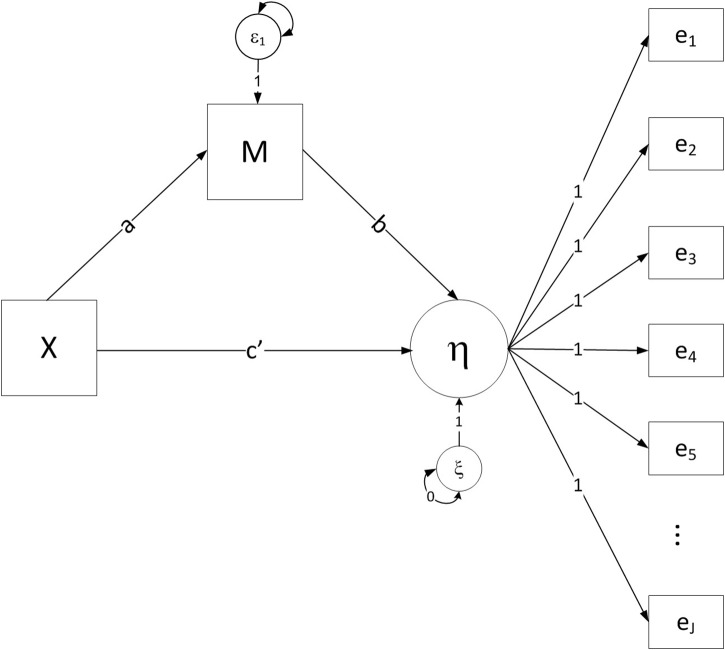
Path diagram for the discrete-time survival mediation model with proportional odds constraint imposed for both the effects of M and X on the event history indicators, where X = the independent variable, M = the mediator variable, η = the latent propensity for event occurrence, and e_1_-e_*j*_ = binary indicators of event occurrence at each time period.

With a single predictor, mediator, and event time, two equations define the DTSM model under a proportional hazard odds assumption that specifies invariant effects of *X* and *M* (see Figure 1).

(1) $$M_{i}=\beta_{01}+aX_{i}+\varepsilon_{1i}$$

(2) $$\mathrm{\eta =logit}\left(P_{h}\left(t_{j}|X_{i},M_{i}\right)\right)=\beta_{02}+c^{\prime }X_{i}+bM_{i}+\varepsilon_{2i}$$

where *P_h_* represents the hazard probability of the event outcome, and η is the logit of the latent propensity for event occurrence^3^. The hazard probability describes the probability of an individual experiencing the event in a particular time interval provided that individual is still event-free at the beginning of the interval. The logit hazard is defined as a linear weighted combination of the predictor *X* and mediator *M*. Indirect effects in the model can be defined in line with tracing rules from the path analysis literature (e.g., Wright, 1921; Alwin and Hauser, 1975), such that a parameter estimate of the mediation effect can be computed by multiplying the *a* coefficient from equation (1) and the *b* coefficient from equation (2). We consider the product of coefficients estimator with bootstrapped confidence limits in our study, as prior methodological work has shown that this is a desirable approach to conducting statistical mediation analysis with a variety of different data types (e.g., Fritz and MacKinnon, 2007; MacKinnon et al., 2007b; Preacher and Hayes, 2008; Taylor et al., 2008)^4^.

The model can be estimated using full-information maximum likelihood procedures as used in conventional logistic regression models (Singer and Willett, 1993; Muthén and Masyn, 2005). This estimation allows for missing data in the form of non-informative right censoring. Individuals with right-censored event times are individuals who are still “event free” at the time they cease to be observed in the study. FIML estimation can incorporate the partial information available about the event times for right-censored individuals into the likelihood function for the sample. Null hypothesis significance testing of mediated effects can be conducted by estimating asymmetric confidence limits for *ab* either via bootstrapping (Efron and Tibshirani, 1993) or the distribution of the product of two random variables (Aroian, 1947; Meeker et al., 1981).

We consider the simplest case of both *X* and *M* as time invariant variables with a time invariant effect for exposition purposes, but more complicated parameterizations of the model are possible. It is straightforward to incorporate time-varying predictors into the DTSM model, as well as to test for evidence of time-varying effects in model estimation. Though the DTSM model can be estimated in an SEM-based mixture-modeling framework to accommodate model extensions (see Fairchild et al., 2015), we limit our scope to the regression-based framework presented in Fairchild et al. (2015) in the interest of simplicity. This facilitates comparison of simulation results to Imai et al. (2010) causal mediation analysis framework. Note however, that in the simplest case as demonstrated here, maximum likelihood estimates of the hazard probabilities in the latent variable parameterization of the model are statistically equivalent to maximum likelihood estimates of the binary event indicators in an observed variable regression approach (see Muthén and Masyn, 2005).

### Potential Outcomes Approach to DTSM

In theory, DTSM implies causal relations among variables in the model. However, as with other mediation models, real-world study design elements will often preclude investigators from drawing causal inferences even in the presence of longitudinal data. Though random assignment of the *X* variable allows the *a* path from the model to be treated as a causal estimate, the *b* and *c’* coefficients cannot be interpreted as causal effects unless there is also random assignment of *M*. Given these limitations, methodologists have worked on developing analytical corrections to improve causal inference in mediation models (Imai et al., 2010; Coffman, 2011; Jo et al., 2011; VanderWeele, 2011). These methods permit one to move beyond associational estimators that consider how variables relate, toward causal estimators that instead evaluate how a given variable of interest causes another (in this case through a third, mediating variable). The frameworks also provide machinery to conduct sensitivity analyses to assess robustness of model effects against omitted confounders (e.g., VanderWeele, 2015). Though there are alternative frameworks available, such as Didelez’s (2018) recent work stemming from the decision theoretic perspective, we focus on the causal mediation approach presented by Imai et al. (2010), which utilizes counterfactuals and is an extension of the potential outcomes framework originally presented by Rubin (1974).

Suppose X is a binary variable, with X = 1 for treatment and X = 0 for control. Imai et al. (2010) define the causal mediation effect based on the potential outcomes framework as:

(3) $$\delta_{i}(t)\equiv Y_{i}(t,M_{i}(1))-Y_{i}(t,M_{i}(0)),\;for\;t=0,\;1$$

where δ(x) represents the difference between the two potential outcomes, changing the mediator from *M*_i_(1) to *M*_i_(0) while holding the treatment constant at *t*. This quantity has also been termed the *natural indirect effect* in the literature. Note that for *M*_i_(1) and *M*_i_(0) only one of the quantities will actually be observed, and thus unit-level causal effects are not identifiable. If the unit participates in the control group _(*X*_*i*_=0)_, then we will observe *M*_i_(0) but not *M*_i_(1). In contrast, if the unit participates in the treatment group _(*X*_*i*_=1)_, then we will observe *M*_i_(1) but not *M*_i_(0). Accordingly, δ(0) reflects how the outcome would change if the mediator value was adjusted from the value that was observed under the control group *M*_i_(0) to the value that would be observed if the participant was assigned to the treatment group *M*_i_(1), and δ(1) reflects how the outcome would change if the mediator value was adjusted from the value that was observed under the treatment group *M*_i_(1) to the value that would be observed if the participant was assigned to the control group *M*_i_(0).

The average causal mediation effect (*ACME*) across participants in a sample is identifiable and given by:

(4) $${\bar{\delta }}_{i}(t)\equiv Ε\left(\delta_{i}(t)\right)\equiv Ε\left(Y_{i}(t,M_{i}(1))-Y_{i}(t,M_{i}(0))\right),\;for\;t=0,\;1$$

Under the sequential ignorability assumption, we can rewrite the equations (1) and (2) as:

(5) $$Ε(M_{i}|X_{i})=\beta_{01}+aX_{i}$$

and

(6) $$Ε(\log it(P_{h}(t|X_{i},M_{i})))=\beta_{02}+c^{\prime }X_{i}+bM_{i}$$

Assuming that there is no interaction between the treatment and the ACME, the ACME is identified and given by $\bar{\delta }$(1) = $\bar{\delta }$(0) = ab. In logit form, the product of coefficients method is equivalent to the causal mediated effect under the potential outcomes framework.

## Methods

To assess the statistical performance of the DTSM model, we conducted a Monte Carlo simulation study. Specifically, we conducted an experiment in which we had knowledge of population parameters by random sampling from probability distributions specified *a priori*. With access to these true parameter values, we defined and evaluated different statistical properties of model estimates. We conducted two variants of the study to demonstrate equivalency of the mediated effect estimates across the product of the coefficients and potential outcomes frameworks. The first study variant was conducted in Mplus Version 6.11 (Muthén and Muthén, 1998–2012) to estimate the product of the coefficients approach in a conventional, observed variable, linear structural model as defined by equations (1) and (2). The second study variant was conducted in R (R Core Team, 2013) using the “mediation” R package for Causal Mediation Analysis Tingley et al. (2014) to estimate the *ACME* as defined above. The latter approach proceeded in two steps. First, we specified the model for the conditional distribution of the mediator *M* given predictor variable *X* and fit a least squares regression through the linear model function. We also fit a model for the conditional distribution of the outcome *Y* given predictor variable *X* and mediator *M* by specifying a generalized linear mixed effect model, with a logit link to model the discrete-time survival data via a random effect component. After fitting the mediator and outcome models, we specified the fitted objects as well as the names of the treatment and mediating variables to the ‘mediate’ function to compute the estimated ACME.

### Monte Carlo Study Design

#### Data Generation

We generated values of the exogenous *X* variable for the simulation studies with the Mersenne Twister pseudorandom number generator (Matsumoto and Nishimura, 1998). The default random number generator in R 2.13.1, the Mersenne Twister algorithm constructs a generally uniform distribution of data with a period of 2^(19937-1)^. Formal tests of statistical randomness have shown that the number sequences the Mersenne Twister generates are sufficiently random. We considered the DTSM model with a dichotomous *X* variable^5^, a specified that the data be randomly sampled from a binomial Bernoulli distribution, *B(1,0.5)*, with probability mass function given by:

(7) $$p\left(x\right)=\{\begin{array}{cc} p^{x}{\left(1-p\right)}^{1-x} & x\in \left\{0,1\right\}\\ 0 & x\notin \left\{0,1\right\} \end{array}$$

We generated data for the *M* variable using equation (2), such that *M* was defined by a linear regression on *X* with β_01_ = 0 and ε_1_ randomly drawn from a standard normal distribution, *N(0,1)*. We generated event histories under a proportional hazard odds model with a constant baseline hazard probability as given by:

(8) $$\mathrm{Pr}\left(e_{ji}|X_{i},M_{i}\right)=\frac{1}{1+\exp\left(-\left(\beta_{02}+c^{\prime }X_{i}+bM_{i}\right)\right)}$$

where β_02_ was fixed to values that corresponded to specific baseline hazard probability values, e.g., β_02_ = -2.94 corresponds to a baseline hazard probability of 0.05. Using a different random seed for each time interval, we randomly sampled values *y_ij_* from a standard logistic distribution, *L(0,1)*, with probability density function given by:

(9) $$f(y)=\frac{\exp\left(-y\right)}{{\left(1+\exp\left(-y\right)\right)}^{2}}$$

If _*y_ij_*>−(β_02_+*c*′*X*+*bM*)_, then *e_ji_* = 1, otherwise *e_ji_* = 0.

After all *e_ji_* values had been generated, values were recoded such that if, for a given case *i*, the first *e_ji_* = 1 occurred at *j=m*, all remaining e_ji_ (i.e.,e_(m+1)i_,…,e_Ji_) were coded as missing, to yield non-informative fixed right-censoring at the end of the final time period.

#### Simulation Parameters

We varied 6 factors in the Monte Carlo study (see Table 1). We considered two variants of the number of measurement occasions associated with the event outcome to investigate how waves of measurement may affect DTSM parameter estimates. We also compared three different sample size conditions to ascertain how performance of the DTSM model fared across a range of sample sizes observed in the social sciences, and evaluated a variety of effect sizes for the *a, b*, and *c’* parameters in the DTSM model to observe how the magnitude of different relations influenced properties of the *ab* parameter estimate in both full mediation and partial mediation contexts. Note that values of the *b* and *c’* parameters reflect partially standardized odds ratios. Finally, we investigated two different baseline hazard conditions to assess in what ways baseline risk impacted performance of the DTSM model. Crossing all levels of each of these factors in a full factorial design yielded 384 unique experimental conditions. Replicating the model estimation process R = 500 times (e.g., MacKinnon et al., 1995; Nylund et al., 2007; Beyersmann et al., 2009) for each unique parameter combination created a sample of 192,000 datasets for analysis. A 6-way 2 × 3 × 4 × 4 × 2 × 2 ANOVA was conducted on the data to analyze the influence of each factor, and all possible interactions among the factors, on outcome variables under study. Due to the extremely large sample size of the experiment and corresponding inflation of statistical significance, we examined the practical significance of effects via η*^2^* to determine whether a given factor or interaction was meaningful. Specifically we interpreted the effect if η*^2^* ≥ 0.01, indicating that at least 1% of the variance in a given outcome was attributable to the effect of interest (Cohen, 1988).

**Table 1:** Simulation factors and corresponding levels of each factor.

Factor	Levels
Time intervals (*J*)	4, 8
Sample size (*n*)	250, 500, 1000
Parameter effect size	
*a* path	0, 0.14, 0.39, 0.59
*b* path	1, 1.5, 2, 4
*c’* path	1, 1.5
Baseline hazard	0.05, 0.2^∗^


*^∗^Corresponding to β_*02*_ = *2.944,1.386*.*

#### Simulation Outcome Measures

We evaluated four statistical properties to assess performance of the DTSM model in the simulation study. First, we looked at the accuracy of parameter estimates by assessing the mean relative bias of the *ab* estimator. We defined mean relative bias by ascertaining the average deviation of the estimator’s expected value from the true population parameter as a proportion of the true value in each unique parameter combination.

(10) $$\hat{RB}\left(\theta \right)=\frac{\hat{E}(\hat{\theta })-\theta }{\theta }$$

where

(11) $$\hat{E}\left(\hat{\theta }\right)=\frac{1}{R}\overset{R}{\underset{r=1}{\sum }}\left({\hat{\theta }}^{\left(r\right)}\right)$$

${\hat{\theta }}^{\left(r\right)}$ refers to the sample estimate of $\hat{a}\hat{b}$ in a given dataset *r*, θ refers to the population value of *ab* in the experimental condition, *r* refers to the replication number, and R is the total number of replications. Values of relative bias ≤0.10 were considered desirable (Muthén et al., 1987). For those parameter combinations where the true value of *ab* was zero and relative bias was therefore undefined, we evaluated accuracy of parameter estimates by simply assessing the magnitude of unstandardized bias for the condition:

(12) $$\hat{B}\left(\theta \right)=\hat{E}\left(\hat{\theta }\right)-\theta$$

We then examined the mean squared error (*MSE*) as a measure of overall accuracy and precision for the *ab* estimator in the DTSM model:

(13) $$\hat{MSE}\left(\theta \right)=\frac{1}{R}\sum_{r=1}^{R}\left[{\left({\hat{\theta }}^{\left(r\right)}-\theta \right)}^{2}\right]=\hat{var}\left(\hat{\theta }\right)+{\left(\hat{B}\left(\theta \right)\right)}^{2}$$

where

(14) $$\hat{var}\left(\hat{\theta }\right)=\frac{1}{R}\sum_{r=1}^{R}\left[{\left({\hat{\theta }}^{\left(r\right)}-\hat{E}(\hat{\theta })\right)}^{2}\right]$$

Though often used as a comparative metric across two or more estimators (where the estimator with the smallest MSE is supported), we analyzed the metric in an absolute sense here to accompany our evaluation of bias in investigating how the simulation factors impact variability in the parameter estimates. As with both measures of bias, smaller values of MSE are desirable.

Finally, we evaluated statistical power and Type 1 error rates to evaluate accuracy of hypothesis testing of the *ab* estimate in the DTSM model. Specifically, we conducted significance testing by using the non-parametric percentile bootstrap to construct asymmetric confidence limits for the mediated effect. A resampling technique, the percentile bootstrap forms the 95% asymmetric confidence limits by empirically constructing the *ab* sampling distribution from the data and identifying the 2.5th and 97.5th quantiles of the distribution. The percentile bootstrap constructs an empirical sampling distribution of *ab* by taking repeated draws with replacement of size=*n* from the data and estimating $\hat{a}\hat{b}$ in each drawn sample. By using the data as a pseudo-population and creating a large number of bootstrap samples in which the parameter is estimated, the resampling technique effectively approximates the sampling distribution of the test statistic. In the studies conducted here, we utilized *n* = 1000 bootstrap draws (e.g., Fisher et al., 2016; Chowdhury et al., 2017; Fang et al., 2019).

For experimental conditions where the true value of *ab* was non-zero, we defined power as the proportion of simulation replications where the 95% asymmetric confidence interval did not include zero. We evaluated the results against the nominal *1-β* = 0.80 criterion (Cohen, 1988), such that the statistical test was characterized as underpowered in conditions where empirical power was <0.80. For experimental conditions where the true value of *ab* was zero, we defined Type 1 error as the proportion of simulation replications where the 95% asymmetric confidence interval did not include zero. We evaluated results against the nominal α = 0.05, such that inflated Type 1 error rates were identified for those conditions where empirical *α* > 0.05.

## Results

### Accuracy and Precision of Parameter Estimates

Because results across the *ab* and ACME estimators were either identical or only differed in the third decimal place for all effects, we just plot results associated with the *ab* estimator. We report both estimates in the text when they differ. When *X* is binary and *M* is a continuous variable, mean relative bias of the *ab* estimator and ACME in the DTSM model (when *ab≠*0) were both 0.013, indicating that parameter estimates were within 1.3% of the true population values across simulation conditions. There were no main effects or interactions of practical significance on the relative bias outcome. In those parameter combinations where the true value of *ab* = 0 or ACME = 0, mean unstandardized bias of the estimators was 0.00007. There were no main effects or interactions of practical significance on the unstandardized bias outcome. These results indicate that the *ab* estimator and ACME in the DTSM model are unbiased when X is binary and *M* is continuous.

**FIGURE 2:**
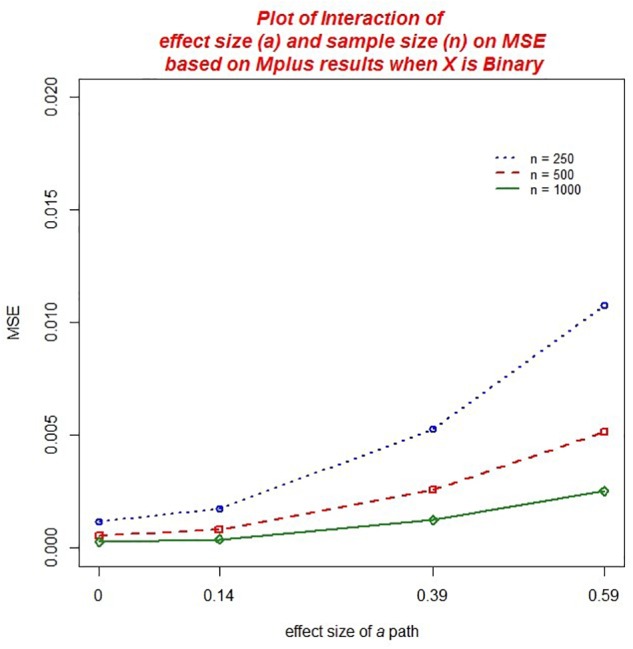
Two-way interaction of sample size and the magnitude of *a* on MSE.

**FIGURE 3:**
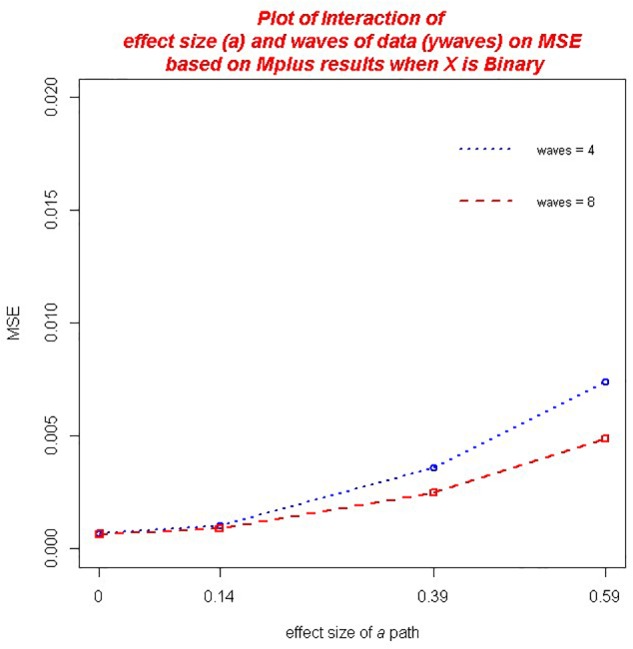
Two-way interaction of number of waves of data collected and the magnitude of *a* on MSE.

**FIGURE 4:**
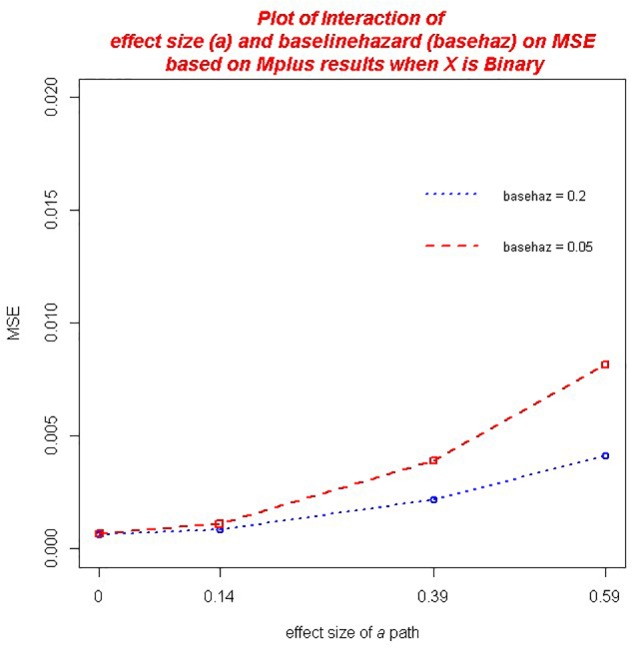
Two-way interaction of baseline hazard and the magnitude of *a* on MSE.

With regard to *MSE* across both the *ab* and ACME estimators, there were several practically significant main effects and interactions. The largest effect, that accounted for 20.9% of the variance in MSE, was a main effect of the size of the *a* path, η*^2^* = 0.209. We also found significant main effect of sample size, η*^2^* = 0.100 as well as the baseline hazard, η^2^ = 0.025. However, there were significant interactions associated with these variables that warrant further discussion. First, there was a practically significant two-way interaction of sample size and the magnitude of *a*,η*^2^* = 0.065 (*ab*) and η^2^ = 0.066 (ACME). The decrease in MSE associated with larger sample size was of greater magnitude for those conditions with a larger *a* path (see Figure 2). We also found a significant interaction of the number of waves of data collected and the size of the *a* path, η*^2^* = 0.011 (*ab*) and η*^2^* = 0.010 (ACME). Here the increase in MSE associated with an increase in the magnitude of the *a* path was mitigated by increasing waves of data collected (see Figure 3). That is, the increase in MSE with increasing size of the *a* coefficient was more negligible with 8 waves of data than with 4 waves of data. In addition, we detected a significant interaction of the baseline hazard and the size of the *a* path, η*^2^* = 0.027 (*ab*) and, η^2^ = 0.028 (ACME). The increase in MSE associated with an increase in the magnitude of the *a* path was moderated by the size of the baseline hazard (see Figure 4). When the baseline hazard was greater, the increase in MSE associated with the *a* path was smaller.

**FIGURE 5:**
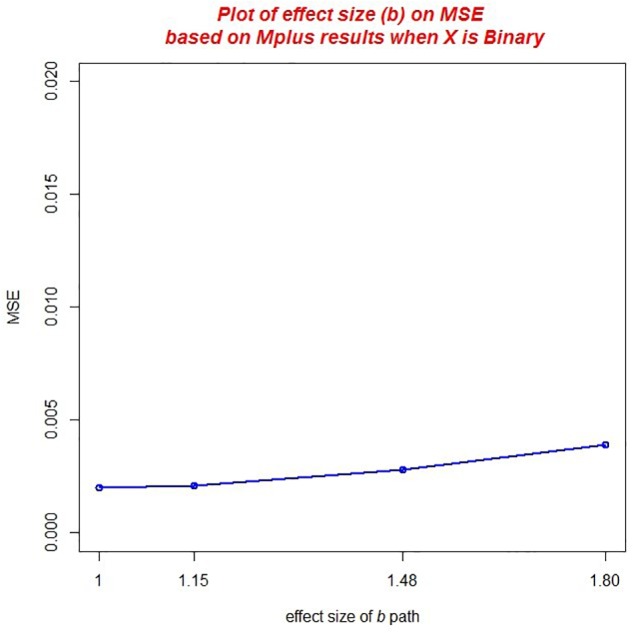
Main effect of the effect size of the *b* path on MSE.

Finally, there was a practically significant main effect of the effect size of the *b* path, η*^2^* = 0.025 (*ab*) and η^2^ = 0.024 (ACME). MSE of the mediated effect increase with increasing effect size of the *b* path (see Figure 5). Across both studies, average MSE was 0.002 for conditions that had a *b* path equal to 1.00 but increased to an average MSE of 0.004 when the *b* path was equal to 1.8.

### Power and Type I Error

The mean Type I error rate for the *ab* estimator in the DTSM model with binary *X* and continuous *M* was α = 0.043; similarly, the mean Type I error rate for the ACME was α = 0.039. There were no practically significant main effects or interactions among simulation factors of practical significance on the outcome. Evaluated against the nominal α = 0.05 criterion, the *ab* estimator and ACME in the DTSM model had acceptable Type I error rates across parameter combinations in these simulation studies.

There were several practically significant main effects and two-way interactions on the power to detect effects in the DTSM model. Specifically, there were significant main effects of sample size, η*^2^* = 0.077 (*ab*) and η*^2^* = 0.075 (ACME), the baseline hazard, η*^2^* = 0.015 (*ab*) and η*^2^* = 0.014 (ACME), the size of the *b* path, η*^2^* = 0.250 (*ab*) and η*^2^* = 0.257 (ACME), and the size of the *a* path, η*^2^* = 0.024 (*ab*) and η*^2^* = 0.026 (ACME). However, these effects should be interpreted in the context of their respective, practically significant interactions.

**FIGURE 6:**
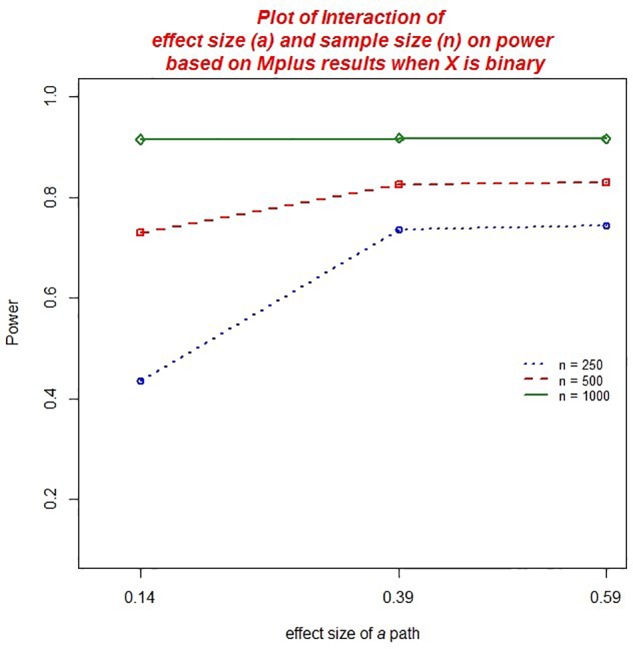
Two-way interaction between sample size and the size of the *a* path on power.

**FIGURE 7:**
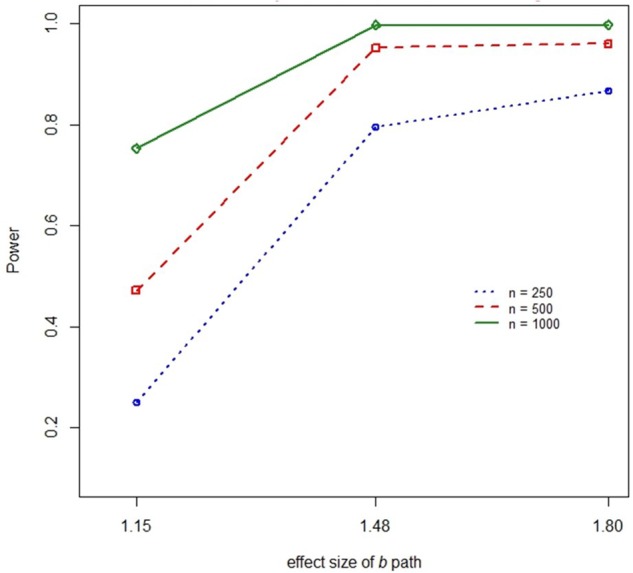
Two-way interaction between sample size and the size of the *b* path on power.

We found a practically significant two-way interaction between sample size and the size of the *a* path, η*^2^* = 0.021 (*ab*) and η*^2^* = 0.020 (ACME), as well as between sample size and the *b* path, η*^2^* = 0.027 (see Figure 6, 7). Power to detect *ab* increased with increasing sample size but this was moderated by the impact of the effect size of the *a* or *b* coefficient. The increase in power associated with increasing sample size was most marked when the size of *a* or *b* was small.

**FIGURE 8:**
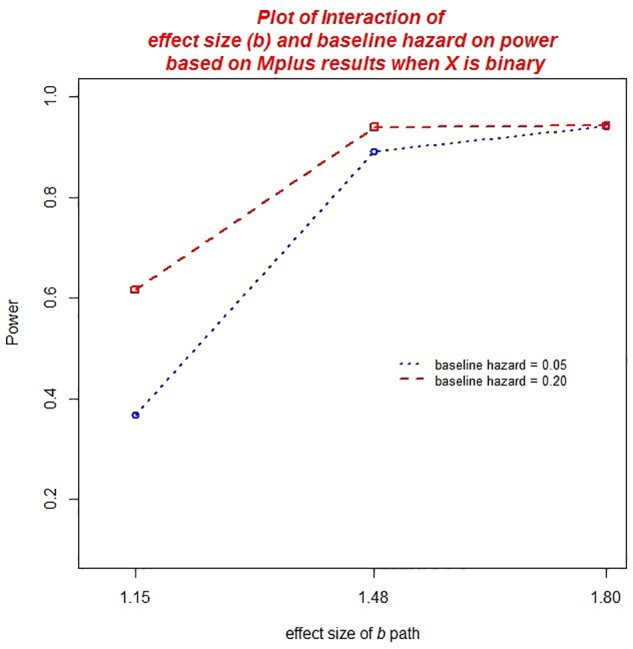
Two-way interaction between baseline hazard and the size of the *b* path on power.

We also noted a practically significant two-way interaction between the baseline hazard and the size of the *b* path, η*^2^* = 0.017 (see Figure 8). In general, greater power was associated with a baseline hazard, however, this effect was moderated by the size of the *b* path. In conditions in which the *b* path was small, power rates were more disparate in conditions with a baseline hazard of 0.05 vs. 0.20. As the effect size of *b* increased, power rates in conditions with baseline hazard of 0.05 vs. 0.20 were more similar.

## Discussion

Discrete-time survival mediation analyses can strengthen behavioral research by improving one’s understanding of predictors of discretized periods of risk over time as well as further illuminating through what mechanisms different predictors impact the timing of an event. Further, DTSM analyses can be used to assess the impact of prevention or intervention programs on time-to-event outcomes. By incorporating the DTSM model into program evaluation efforts, researchers can enhance their understanding of how prevention or intervention programs achieve (or fail to achieve) effects on time-to-event outcomes.

This article evaluated the statistical performance of a DTSM model originally presented by Fairchild et al. (2015) in a Monte Carlo simulation study. It was shown that the conventional product of coefficients estimator and the *ACME* yielded comparable model results in the simulation conditions under study. Results showed favorable statistical properties of the model across a variety of different conditions. Specifically, MSE and Type 1 error for the model were tolerable across a range of different sample sizes, effect sizes, baseline hazard conditions, and number of measurement waves. Adequate power to detect mediation effects, however, required that effect sizes of the model parameters be medium or large. Note that considering the performance of a *statistical test* for mediation inherently involves null hypothesis significance testing, which is not without limitations. Given these limitations, and in line with other authors (e.g., Sullivan and Feinn, 2012), we encourage readers to report effect size estimates in any statistical model to assess the magnitude of an effect under study. Alternatively, researchers may consider different strategies entirely to investigate mediation effects (e.g., design approaches; Pirlott and MacKinnon, 2016; or Bayesian analyses; Enders et al., 2013; Miočević et al., 2018).

### Limitations and Model Extensions

As this was the first examination of the DTSM model, we considered the simplest case of a single mediator model with time-invariant predictors that satisfied a proportional hazard odds assumption. These constraints are necessarily a limitation of the study that could be explored in subsequent research. Accommodating time-varying predictors and non-proportionality in the DSTMed model involves multiple *a, b*, and *c’* paths, which necessarily introduces additional issues in estimating the mediated effect.

Future work might consider evaluating different latent variable model extensions of the DTSM model that permit examination of more complicated longitudinal processes. One such example is a finite mixture model that examines joint event history and growth processes (e.g., Malone et al., 2012). Such models permit the examination of reciprocal effects across the two processes, and may provide a good foundation for exploring multifaceted developmental theories. The estimation of non-parametric frailties to model unobserved heterogeneity in the survival process is also possible. Such random factors have been shown to be important in continuous survival models (e.g., Henderson and Oman, 1999), and may be particularly relevant to multivariate survival models where recurring events and/or competing risks are of interest.

### Recommendations and Concluding Remarks

The utility of evaluating mediation effects spans across research domains, as such analyses strengthen prevention science and etiological research more generally. In order for the analyses to be maximally useful however, it is critical to employ methodology appropriate for the data under investigation (e.g., logistic or probit models for categorical outcomes; multilevel models for clustered data). The DTSM model presented in this article intends to facilitate the investigation of mediation effects with discrete-time survival data. By evaluating discrete-time mediation effects, we can improve our understanding of discretized periods of risk for myriad behavioral outcomes, as well as target prevention and intervention work more effectively. Interested researchers may consult Fairchild et al. (2015) for applied example syntax and a detailed interpretation of model parameters in the substantive context, for the model specification evaluated here^6^.

Simulation results indicate that parameter estimates of mediation in the DTSM model were statistically accurate and precise across the range of examined conditions. Type 1 error rates of the *ab* estimator and ACME were also tolerable in the conditions studied. Thus, substantive researchers can feel confident in using the DTSM model to make statistical inferences in circumstances where the model is adequately powered. With binary *X* variables, we cannot advocate that the model be used when the relation between *X* and *M* is posited to be small in effect, however. Though it may be possible that the model achieves adequate power with a small effect size of *a* in sample sizes larger than *n* = 1000, no such conditions were considered in this study precluding any comment on the matter.

Finally, as noted by Pratschke et al. (2016), a linear specification of the *DTSM* model using an applicable link function (such as the logit) allows for a causal interpretation of *ab* in this context. That is, the product of coefficients will yield an estimate of the natural indirect effect on a logit scale, lending support for *ab* as a viable estimator of mediation in linearized discrete-time survival models when there are no interaction effects. When incorporating non-linear relationships into the model, however, we encourage researchers to estimate ACME effects. The product of coefficients diverges from the causal estimator in this case, and *ab* no longer retains a causal interpretation (a limitation of the estimator). Fortunately, both methods to estimate mediation effects are readily available in a wide variety of software packages.

## Author Contributions

AF designed, wrote, and approved all contributions to the study. CC ran all analysis for the work. HM and DS helped to revise the study. AG ran earlier analyses that contributed to the work. KM helped to design the study.

## Conflict of Interest Statement

The authors declare that the research was conducted in the absence of any commercial or financial relationships that could be construed as a potential conflict of interest.

## Footnotes

1 The focus of this paper is on mediation models, where a discrete-time onset variable serves as the outcome. Note that there are other types of mediation models where putative mediators can take the form of a discrete-time onset variable. Kilburn et al. (2018) examined one such model in the context of a randomized controlled trial to understand the impact of a conditional cash transfer intervention on reducing intimate partner violence for young women in South Africa.

2 Beyond issues associated with statistical power, the causal steps notion that there needs to be a significant overall relation of the predictor and outcome variables for mediation to exist is fallible from a variety of additional perspectives. Though testing the overall relationship between a predictor and outcome is important in any research study, it is a separate test from mediation and indirect effects can exist in the absence of a significant overall effect. This might occur in multiple mediator models where different mediated effect pathways have varying signs or when the sign of the mediated effect is opposite in value from the direct effect, yielding inconsistent mediation models.

3 Note: we use a latent underlying response variable parameterization, with latent variable η, as estimated in Mplus.

4 Previous work has noted the fallibility of estimating mediation with a difference in coefficients approach for both categorical and time-to-event outcomes (e.g., Lapointe-Shaw et al., 2018).

5 We specified a dichotomous X variable to align with a randomized controlled trial scenario where there would be treatment and control groups.

6 A simulation code template file for the current study is provided for interested readers in the Supplementary Materials to this manuscript.

## Supplementary Material

DATA SHEET S1 Partial R Code for Simulation Study.
