# Supplementary material for: Evaluating a Method to Estimate Mediation Effects With Discrete-Time Survival Outcomes
Source: Front Psychol. 2019 Apr 5;10:740. doi: 10.3389/fpsyg.2019.00740 (PMC6460901; doi:10.3389/fpsyg.2019.00740)
Supplement: DATA SHEET S1 — Partial R Code for Simulation Study. [file Data_Sheet_1.PDF]

```
####################simulation code for estimating ACME####################
library("mediation")
library("mi")
trace("mediate",edit=TRUE)  # comment the link transformation part #


setwd("C:/Users/caic/Documents/Chao Cai/DTSMM Simulation")
parent <- getwd()

ywavparm <- c(4, 8)

nparam <- c(250, 500, 1000)
aparam <- c(0, 0.14, 0.39, 0.59)
bparam <- c(1, 1.5, 2, 4)
cprmparm <- c(1, 1.5)
bhazparm <- c(0.05, 0.2)

allparmcombos <- expand.grid(yID = 1:length(ywavparm), nID =
1:length(nparam),
      aID = 1:length(aparam), bID = 1:length(bparam),
      cID = 1:length(cprmparm), bhID = 1:length(bhazparm))

absigfunc <- function(lcl2.5, ucl2.5) {
      abSig <- as.integer(NA)
      if (lcl2.5<0 && ucl2.5>0) abSig <- 0 else abSig <- 1
      return(abSig)
      }
      abpopinCIfunc <- function(ll2.5, ul2.5, popab) {
      abPopInCI <- as.integer(NA)
      if (ll2.5<popab && ul2.5>popab) abPopInCI <- 1 else abPopInCI <- 0
      return(abPopInCI)
      }
relbiasfunc <- function(abAveEst, abPop) {
      relbias <- as.numeric(NA)
      if (abPop != 0) relbias  <- as.numeric(((abAveEst - abPop) / abPop))
            else relbias <- as.numeric(NA)
      return(relbias)
      }
powerfunc <- function(PropSigCI, abPop) {
      power <- as.numeric(NA)
      if (abPop != 0) power <- as.numeric(PropSigCI)
            else power <- as.numeric(NA)
      return(power)
      }
typeIerrfunc <- function(PropSigCI, abPop) {
      typeIerr <- as.numeric(NA)
      if (abPop == 0) typeIerr <- as.numeric(PropSigCI)
            else typeIerr <- as.numeric(NA)
      return(typeIerr)
      }
ptm <- proc.time()
for (parmset in 101:200) {
      ywaves <- ywavparm[allparmcombos[parmset,"yID"]]
```

```
      n <- nparam[allparmcombos[parmset,"nID"]]
      a <- aparam[allparmcombos[parmset,"aID"]]
      b <- bparam[allparmcombos[parmset,"bID"]]
      cprime <- cprmparm[allparmcombos[parmset,"cID"]]
      basehaz <- bhazparm[allparmcombos[parmset,"bhID"]]

      timepointsID <- match(ywaves, ywavparm)
      sampsizeID <- match(n, nparam)
      apathID <- match(a, aparam)
      bpathID <- match(b, bparam)
      cpathID <- match(cprime, cprmparm)
      basehazID <- match(basehaz, bhazparm)

      condID <- as.integer(paste(basehazID, cpathID, bpathID,
      apathID, sampsizeID, timepointsID, sep = ""))

      subdir <- paste(parent, "/c", condID, sep = "")
      setwd(subdir)

      nrep=10

      abLevel <- array(NA, dim=c(nrep,7))
      colnames(abLevel) <- c("a.est","b.est","cprime.est","ACME","p-
value","low2.5","up2.5")
      sig <- array(NA, dim=c(nrep,2))
      colnames(sig) <- c("ab_sig","ab_pop_in_CI")

      for (j in 1:nrep) {
      subdir2 <- paste(parent, "/c", condID, "/c", condID, "_x_r", j,".dat",
sep = "")
      tst <- read.table(subdir2)
      n <- nrow(tst)
      tst <- tst[,c(2,4,5,6,7,8)]
      tst2 <- data.frame(id = rep(1:n), x=tst[,1], m=tst[,2], y3=tst[,3],
y4=tst[,4], y5=tst[,5], y6=tst[,6])
      one <- reshape(tst2, varying=c("y3","y4","y5","y6"),
            v.names="y", timevar = "time",
            times=c("y3","y4","y5","y6"),
            direction = "long")
      one.sort <- one[order(one$id),]
      one.sort$y[one.sort$y==-999] <- NA
      D1 <- ifelse(one.sort$time=="y3",1,0)
      D2 <- ifelse(one.sort$time=="y4",1,0)
      D3 <- ifelse(one.sort$time=="y5",1,0)
      D4 <- ifelse(one.sort$time=="y6",1,0)
      pp.data <-
data.frame(id=one.sort$id,D1,D2,D3,D4,x=one.sort$x,m=one.sort$m,y=one.sort$y)

      IMP <- mi (pp.data, n.imp=50, add.noise=FALSE)
      imputed.matrix <- mi.completed (IMP)
      imputedReslt <- array(NA, dim=c(50,7))
            for(i in 1:50)
                  { m1 <- lm(m ~ x, data=imputed.matrix[[i]])
```

```
            imputedReslt[i,1] <- m1$coefficients[2]
            m2 <- glm(y ~ x + m + D1 +D2 + D3, data=imputed.matrix[[i]],
family=binomial)
            imputedReslt[i,2] <- m2$coefficients[3]
            imputedReslt[i,3] <- m2$coefficients[2]
            med.out <- mediate(m1, m2, treat = "x", mediator = "m", sims =
100)
            imputedReslt[i,4] <- med.out$d.avg
            imputedReslt[i,5] <- med.out$d.avg.p
            imputedReslt[i,6] <- med.out$d.avg.ci[1]
            imputedReslt[i,7] <- med.out$d.avg.ci[2]
            }
      otp <- apply(imputedReslt,2,mean)
      abLevel[j,] <- otp

      sig[j,1]<- as.integer(mapply(absigfunc, lcl2.5 = abLevel[j,"low2.5"],
ucl2.5 = abLevel[j,"up2.5"]))
      sig[j,2] <- as.integer(mapply(abpopinCIfunc, ll2.5 =
abLevel[j,"low2.5"], ul2.5 = abLevel[j,"up2.5"], popab = a*log(b)))

      #subdir3 <- paste(parent, "/c", condID, "/Rpackage_x_","c", condID,
"_r", j, ".txt", sep = "")
      #write.table(abLevel[j,] , file = subdir3)
      }
      pop_a <- a; pop_b <- log(b); pop_ab <- a*log(b);
      abRepLevel <- cbind(abLevel, sig, pop_a, pop_b, pop_ab, condID)
      subdir4 <- paste(parent, "/c", condID, "/Rpackage_x_","c", condID,
"_ab_rep-level", ".txt", sep = "")
      write.table(abRepLevel, file = subdir4)
      ave_est <- aggregate(abRepLevel[,"ACME"], list(abRepLevel[,"condID"]),
mean, simplify=TRUE)
      names(ave_est) <- c("condID","AveEst")
      prop_CIsig <- aggregate(abRepLevel[,"ab_sig"],
list(abRepLevel[,"condID"]), mean, simplify=TRUE)
      names(prop_CIsig) <- c("condID","PropCISig")
      prop_CIpop <- aggregate(abRepLevel[,"ab_pop_in_CI"],
list(abRepLevel[,"condID"]), mean, simplify=TRUE)
      names(prop_CIpop) <- c("condID", "PropCIPop")
      abCondLevel <-  merge(prop_CIsig, prop_CIpop, by="condID", all=TRUE,
sort=TRUE)
      abCondLevel <- merge(ave_est, abCondLevel, by="condID", all=TRUE,
sort=TRUE)
      abCondLevel <- cbind(abCondLevel,condID,pop_a,pop_b,pop_ab)
      abCondLevel$RelBias <- as.numeric(mapply(relbiasfunc, abAveEst =
abCondLevel$AveEst, abPop = abCondLevel$pop_ab))
      abCondLevel$Power <- as.numeric(mapply(powerfunc, PropSigCI =
abCondLevel$PropCISig, abPop = abCondLevel$pop_ab))
      abCondLevel$TypeIError <- as.numeric(mapply(typeIerrfunc, PropSigCI =
abCondLevel$PropCISig, abPop = abCondLevel$pop_ab))
      abCondLevel$CIcoverage <- abCondLevel$PropCIPop
      abCondLevel$Bias <- abCondLevel$AveEst - abCondLevel$pop_ab
      abCondLevel$MSE <- mean((abRepLevel[,"ACME"] -
abRepLevel[,"pop_ab"])^2)
```

```
      subdir5 <- paste(parent, "/c", condID, "/Rpackage_x_","c", condID,
"_ab_cond-level", ".txt", sep = "")
      write.table(abCondLevel, file = subdir5)
}
proc.time() - ptm


#####################simulation code for estimating ab#####################

[[init]]

iterators = timepoints sampsize apath bpath cpath basehaz repid ;
timepoints = 1:2 ;
sampsize = 1:3 ;
apath = 1:4 ;
bpath = 1:4 ;
cpath = 1:2 ;
basehaz = 1:2 ;
repid = 1:10 ;

timepointsnames#timepoints = 4 8 ;
sampsizenames#sampsize = 250 500 1000 ;
apathnames#apath = 0 0.14 0.39 0.59 ;
bpathnames#bpath = 1 1.5 2 4 ;
cpathnames#cpath = 1 1.5 ;
basehaznames#basehaz = 0.05 0.2 ;

cpus = 4 ;

filename = "[insert filename of choice.inp]" ;
outputDirectory = "[insert path name of choice]" ;

[[/init]]

TITLE:
      [insert title name of choice];



DATA:
    FILE IS c[insert filename of choice].dat ;

VARIABLE:
    [[timepoints = 1]]
    NAMES ARE rep x1 m2a m2b y3-y6 ;
    USEVARIABLES ARE x1 m2a m2b y3-y6 ;
    CATEGORICAL = y3-y6 ;
    [[/timepoints = 1]]
    [[timepoints = 2]]
    NAMES ARE rep x1 m2a m2b y3-y10 ;
    USEVARIABLES ARE x1 m2a m2b y3-y10 ;
    CATEGORICAL = y3-y10 ;
    [[/timepoints = 2]]
```

```
    MISSING ARE ALL (-999) ;
    CLASSES = class (1) ;

ANALYSIS:
    TYPE = MIXTURE ;
    ESTIMATOR = ML ;
    LINK = LOGIT ; !
    ALGORITHM = EM ;
    BOOTSTRAP = 1000 ;
    PROCESSORS = [[cpus]] ;

MODEL:
    %OVERALL%

    m2a ON x1 (p1) ;
    [m2a] ;
    m2a ;

    [[timepoints = 1]]
    [y3$1-y6$1] ;
    factor BY y3-y6@1 ;
    [[/timepoints = 1]]
    [[timepoints = 2]]
    [y3$1-y10$1] ;
    factor BY y3-y10@1 ;
    [[/timepoints = 2]]
    factor ON x1 m2b (p2-p3) ;
    !factor@0 ;

MODEL CONSTRAINT:
    NEW (ab) ;
    ab = p1 * p3 ;

OUTPUT:
   CINTERVAL (BOOTSTRAP) ;


! NOTE 1:
!   Use different variables for M2 as a DV and M2 as an IV
!   This avoids issues of M2 being treated as latent when it is an IV in the
DTSM
!   (M2 is treated as latent by default when it's the DV in 1st mediation
regression)

! NOTE 2:
!   Referencing factor variance makes Mplus consider this estimate (even if
fixing value)
!   and requires INTEGRATION (b/c latent factor w/ categorical indicators w/
missing data)
!   default is to *not* estimate factor variance (=0) and will avoid invoking
INTEGRATION
```
